# Dual-specificity phosphatase 1 interacts with prohibitin 2 to improve mitochondrial quality control against type-3 cardiorenal syndrome

**DOI:** 10.7150/ijms.90484

**Published:** 2024-01-19

**Authors:** Nanyang Liu, Yanqiu Ding, Hao Zhou, Xing Chang, Long Lou

**Affiliations:** 1 Xiyuan Hospital, China Academy of Chinese Medical Sciences, Beijing, China.; 2 Graduate School, Beijing University of Chinese Medicine, Beijing, China.; 3 Cardiovascular department, Guang'anmen Hospital, China Academy of Chinese Medical Sciences, Beijing, 100053, China.; 4 Kunming Municipal Hospital of Traditional Chinese Medicine, Yunnan, China.

**Keywords:** CRS-3, DUSP1, PHB2, mitochondrial quality control

## Abstract

Type-3 cardiorenal syndrome (CRS-3) is acute kidney injury followed by cardiac injury/dysfunction. Mitochondrial injury may impair myocardial function during CRS-3. Since dual-specificity phosphatase 1 (DUSP1) and prohibitin 2 (PHB2) both promote cardiac mitochondrial quality control, we assessed whether these proteins were dysregulated during CRS-3-related cardiac depression. We found that DUSP1 was downregulated in heart tissues from a mouse model of CRS-3. DUSP1 transgenic (DUSP1*^Tg^*) mice were protected from CRS-3-induced myocardial damage, as evidenced by their improved heart function and myocardial structure. CRS-3 induced the inflammatory response, oxidative stress and mitochondrial dysfunction in wild-type hearts, but not in DUSP1*^Tg^* hearts. DUSP1 overexpression normalized cardiac mitochondrial quality control during CRS-3 by suppressing mitochondrial fission, restoring mitochondrial fusion, re-activating mitophagy and augmenting mitochondrial biogenesis. We found that DUSP1 sustained cardiac mitochondrial quality control by binding directly to PHB2 and maintaining PHB2 phosphorylation, while CRS-3 disrupted this physiological interaction. Transgenic knock-in mice carrying the *Phb2^S91D^* variant were less susceptible to cardiac depression upon CRS-3, due to a reduced inflammatory response, suppressed oxidative stress and improved mitochondrial quality control in their heart tissues. Thus, CRS-3-induced myocardial dysfunction can be attributed to reduced DUSP1 expression and disrupted DUSP1/PHB2 binding, leading to defective cardiac mitochondrial quality control.

## Introduction

With the prolongation of life expectancy and the increasing aging population, the proportion of patients with concurrent cardiovascular and renal diseases is increasing year by year. Acute and chronic heart diseases can directly induce or worsen renal impairment, and vice versa 1. The bidirectional interactions between the heart and the kidneys complicate disease treatment, reduce the quality of life, prolong hospitalization, worsen the prognosis and increase mortality 2, 3. Therefore, the concept of cardiorenal syndrome (CRS) is receiving increasing attention.

Type-3 CRS (CRS-3) is defined as acute kidney injury followed by cardiac injury and/or dysfunction 4. Various pathophysiological processes contribute to the occurrence and development of CRS-3. Fluid retention during acute kidney injury can lead to pulmonary edema 5. Electrolyte disturbances such as hyperkalemia can cause arrhythmias and even cardiac arrest 6. Untreated uremia can impair myocardial contractility by fostering myocardial depressant factor accumulation and pericarditis 7. Metabolic acidosis can promote the progression of right heart failure by inducing pulmonary vasoconstriction, and can also have negative inotropic and proarrhythmic effects 7. In addition, renal ischemia itself can trigger inflammatory mediator production, leukocyte infiltration and apoptosis, leading to inflammation-induced myocardial injury 8. When acute kidney injury progresses and requires renal replacement therapy, rapid changes in fluid volume and electrolyte levels can induce or exacerbate hypertension, arrhythmias and myocardial ischemia 9. However, the molecular pathways underlying CRS-3 are not fully understood, so effective therapeutic approaches are still lacking.

Recent findings have revealed that mitochondrial dysfunction may promote the progression of heart failure during CRS-3 8, 10, 11. Mitochondrial injury is associated with mitochondrial oxidative stress, calcium imbalances, adenosine triphosphate (ATP) shortages and mitochondrial apoptotic pathway activation 12. On the other hand, pharmacological interventions to enhance mitochondrial fitness have been shown to improve heart performance during CRS-3 10, 11. However, mitochondrial function can also be restored through the endogenous defensive mechanism known as mitochondrial quality control, which consists of mitochondrial dynamics, mitophagy and mitochondrial biogenesis 13-15. Mitochondrial quality control can modify the mitochondrial morphology, promote mitochondrial turnover and stimulate mitochondrial metabolism 16, 17. The beneficial effects of mitochondrial quality control on the heart have been widely reported by our laboratory 18 and other colleagues 19-21. However, the influence of mitochondrial quality control on CRS-3 has not yet been described.

Dual-specificity phosphatase 1 (DUSP1, also known as MKP-1) is a critical enzyme involved in cellular signaling pathways 22. Belonging to the family of dual-specificity phosphatases, DUSP1 can (de)phosphorylate threonine/serine and tyrosine residues on target proteins 23-25. We previously demonstrated that DUSP1 is critically important for mitochondrial quality control in cardiac ischemia/reperfusion injury 26 and endotoxemia-related myocardial dysfunction 16. Since DUSP1 is not localized to the mitochondrial surface, its impact on mitochondrial structure and function must be indirect and involve other proteins 16, 27.

Prohibitin 2 (PHB2) is a protein that primarily localizes to the inner mitochondrial membrane 28, where it maintains mitochondrial structure and function 29, 30. In light of its enhancement of mitophagy and mitochondrial dynamics 30, 31, PHB2 has been identified as a novel inducer of mitochondrial quality control in diabetic cardiomyopathy 32 and doxorubicin-induced cardiomyopathy 33. Importantly, phosphorylation of PHB2 can alter its interactions with other proteins, its subcellular localization and its function in mitochondrial dynamics 34, 35. Considering that DUSP1 is a dual-specificity phosphatase, we wondered whether it might promote mitochondrial quality control by altering PHB2 phosphorylation. In this study, we explored whether cardiac dysfunction during CRS-3 was associated with dysregulated DUSP1 activity, PHB2 phosphorylation and mitochondrial quality control.

## Materials and Methods

### Animals and CRS-3 model

All animal procedures were performed according to guidelines and protocols approved by the Institutional Animal Care and Use Committee of Xiyuan Hospital, China Academy of Chinese Medical Sciences. DUSP1*^Tg^* 16 and *Phb2^S91D^* knock-in mice 36 on a C57BL/6 background were provided by Prof. Hao Zhou in Chinese PLA General Hospital. Mice (8-10 weeks old) were maintained under pathogen-free conditions on a 12-hour light/dark schedule with *ad libitum* access to water and a chow diet.

For CRS-3 model generation, anesthesia was administered via intraperitoneal injection of 1% pentobarbital at a dose of 80 mg/kg body weight. The hair was removed from both sides of the mouse's back, and the area was disinfected with iodine. A 1-cm incision was made through the skin and muscle beside the spine to expose the left kidney. The renal artery and vein on both sides of the kidney were carefully separated, and the blood vessels were promptly clamped using artery forceps. The clamping time was noted, and the procedure was repeated for the other kidney. After the procedure, the mouse was placed on a warming pad at 37 °C. After 30 minutes of clamping, the vascular clamp was released, and the kidney was observed to ensure that it was rapidly changing color from purplish-brown to red. To prevent dehydration, 1 mL of saline was administered. The incisions on both sides were closed in layers using sutures. The mouse was kept on the warming pad for five to six hours so that its recovery could be monitored, and then it was returned to the animal facility. Samples were collected at various time points as per the experimental schedule.

### Echocardiography

A Fujifilm VisualSonics Vevo 2100 Ultra High-Frequency Imaging Platform was used for the functional analysis. During echocardiography, the mice were anaesthetized with 1.5% isoflurane, with continuous monitoring of the electrocardiogram, respiratory rate and body temperature. The LVEF, FS and ventricular volumes were measured in parasternal long-axis view using the Vevo LV-Trace function. The wall thickness was measured in the parasternal long- and short-axis M-Modes. Aortic valve regurgitation was imaged using color-Doppler mode in the parasternal long-axis and suprasternal views.

### Transmission electron microscopy

Transmission electron microscopy was used to monitor the myocardial ultrastructure, as described previously 37. Briefly, a freshly collected sample from the apex of the mouse heart was dissected into 1-mm^3^ sections, immediately fixed with 2% glutaraldehyde in 0.1 M phosphate-buffered saline, and then fixed with 1% osmium tetroxide. After the samples were dehydrated in ethanol and embedded in Epon resin, ultrathin sections were prepared and counterstained with uranyl acetate and lead citrate. The stained sections were examined under a transmission electron microscope (JEOL1230). The mitochondrial number was counted in a total of 10 images per heart (x12,000 magnification, n=3 hearts per group).

### Hematoxylin and eosin (H&E) staining

Heart tissues were fixed in 10% formalin (Thermo Fisher Scientific, Agawam, CA, USA) and stained with H&E. Images were captured on a Panoptiq Digital Slide Imaging System.

### Immunofluorescence

Heart tissue sections (4 µm) and cells were fixed with phosphate-buffered 4% paraformaldehyde (0335, Carl Roth) for 10 minutes. Antibodies were diluted in Dako antibody diluent (S202230-2, Agilent Technologies). For immunofluorescence staining, the sections were incubated with primary antibodies overnight at 4 °C. After the sections were washed three times with phosphate-buffered saline (A0964.9050, VWR International, Radnor, PA, USA), they were incubated with fluorophore-conjugated secondary antibodies for one hour at room temperature. The sections were mounted with Dako fluorescence mounting medium (S302380-2, Agilent Technologies).

### Cardiomyocyte isolation

Ventricular cardiomyocyte isolation was performed as previously described 38. Mice were anesthetized with 5% isoflurane and then killed via cervical dislocation. Their hearts were rapidly extracted and placed in Tyrode's solution containing 140 mM NaCl, 6 mM KCl, 1 mM MgCl_2_, 1 mM CaCl_2_, 10 mM glucose and 10 mM 4-(2-hydroxyethyl)-1-piperazineethanesulfonic acid (HEPES), adjusted to pH 7.4 with 2 mM NaOH. After aortic cannulation, the hearts were placed in a Langendorff system, perfused with Tyrode's solution for five minutes, and then perfused for five minutes with a low-Ca^2+^ oxygenated solution containing 120 mM NaCl, 5.4 mM KCl, 5 mM MgSO_4_, 5 mM sodium pyruvate, 20 mM glucose, 20 mM taurine, 10 mM HEPES, 5 mM nitrilotriacetic acid and 0.04 mM CaCl_2_, adjusted to pH 6.96 with 2 mM NaOH. Finally, the hearts were perfused for 10 minutes with an enzyme solution containing 120 mM NaCl, 5.4 mM KCl, 5 mM MgSO_4_, 5 mM sodium pyruvate, 20 mM glucose, 20 mM taurine, 10 mM HEPES and 0.2 mM CaCl_2_, pH 7.4, with collagenase type 2 (1 mg/mL; Worthington) and hyaluronidase (0.6 mg/mL; Sigma-Aldrich). Left ventricular cardiomyocytes were plated on laminin-coated dishes and allowed to attach to the dish/coverslip for at least 45 minutes before experiments.

### Analysis of contraction/relaxation kinetics

Contractile kinetics were analyzed at the indicated time points in a monolayer of cultured cardiomyocytes. In brief, cells were seeded onto Matrigel-coated 96-well plates at a density of 100,000 cells per well in high-glucose Dulbecco's modified Eagle's medium supplemented with 10% fetal bovine serum for 24 hours. Contraction/relaxation movement was recorded every 56 milliseconds for 16 seconds using an inverted microscope (Olympus CellR). During the recording process, the temperature was kept at 37 °C and the atmosphere was maintained at 5% CO_2_. Data were analyzed in ImageJ using the 'MuscleMotion' tool, as we previously described 39.

### Mitochondrial membrane potential detection

For mitochondrial membrane potential quantification, cardiomyocytes were incubated in medium containing 10 μgl/L JC-1 (T3168, Thermo Fisher) at 37 °C for 10 minutes. Cells were analyzed using a confocal laser scanning microscope 40.

### ROS measurements

ROS were measured as we described previously 37. In brief, cells were incubated with DCFH-DA (5 μM, Invitrogen, Eugene, OR, USA), and the fluorescence intensity was measured with excitation at 510 nm and emission at 580 nm using a confocal laser scanning microscope. The mean level was set at control = 1, and the fold-increase was calculated for each measurement 41.

### RNA extraction and qPCR

Total mRNA was extracted from mouse heart tissues with a Quick RNA miniprep kit (Zymo Research, Irvine, CA, USA) according to the manufacturer's instructions. Then, qPCR was performed to detect mRNA expression using SYBR Green Master Mix (Applied Biosystems, Foster City, CA, USA) in a QuantStudio™ 6 Flex system (Applied Biosystems). The expression of each gene was normalized to that of 18S rRNA 42. Thermal cycling was conducted with an initial hold period at 95 °C for 10 minutes, followed by 40 cycles of a three-step PCR program at 95 °C for 15 seconds, 60 °C for 1 minute and 72 °C for 30 seconds.

### Cell culture and siRNA knockdown of *DUSP1*

HL-1 cells were used for the *in vitro* studies. The HL-1 cell line was purchased from the JCRB Cell Bank. Cells were cultured in Dulbecco's modified Eagle's medium (Gibco, Grand Island, NY, USA) with 10% fetal bovine serum (Gibco), 100 U/mL penicillin and 100 g/mL streptomycin (Gibco) at 37 °C in a humidified incubator containing 5% CO_2_. For the knockdown experiments, cells were seeded in 24-well plates in triplicate for 48 hours. *DUSP1* siRNAs (#sc-35937; Santa Cruz Biotechnology, Inc.) were transfected into the HL-1 cells with Lipofectamine™ 3000 reagent, as described in our previous study 43.

### Protein extraction and Western blot analysis

Mouse heart tissues and cells were homogenized in M-PER™ Mammalian Protein Extraction Reagent (Cat #78501, Thermo Fisher Scientific, Waltham, MA, USA) containing Halt™ Protease Inhibitor Cocktail (Cat #78429, Thermo Fisher Scientific). The protein concentrations were determined with a Pierce™ Microplate BCA Protein Assay Kit (Cat #23252, Thermo Fisher Scientific) according to the manufacturer's instructions 44, 45. For Western blotting, the extracted proteins were boiled in Laemmli sample buffer (2X; Cat #1610737, Bio-Rad Laboratories, Hercules, CA, USA) for denaturation, and then were electrophoretically separated on sodium dodecyl sulfate polyacrylamide gels (Cat #M00654, GenScript, Piscataway, NJ, USA) and transferred onto nitrocellulose membranes using a Trans-Blot Turbo Transfer System and RTA Transfer Packs (Cat #1704271, Bio-Rad Laboratories). The membranes were blocked with 10% non-fat dry milk in Tris-buffered saline containing 0.05% Tween-20 for one to two hours at room temperature, and then were incubated with primary antibodies (summarized in Table S5) at 4 °C overnight. The membranes were then incubated with horseradish peroxidase-conjugated secondary antibodies (1:5000; Jackson ImmunoResearch Laboratories Inc., West Grove, PA, USA) at room temperature for one hour. After appropriate washing, the membranes were developed with Clarity^TM^ Western ECL Substrate (Cat #170-5061, Bio-Rad Laboratories) or Clarity^TM^ Max Western ECL Substrate (Cat #170-5062, Bio-Rad Laboratories). The Western blots were quantified using ImageJ software (Version 1.51n, National Institutes of Health, Bethesda, MD, https://imagej.nih.gov/ij), as previously described 46.

### ELISA

A Mouse BNP ELISA kit (#P40753, RayBiotech Life, Inc.), Mouse lactate dehydrogenase (LDH) ELISA kit (#SEB864Mu, CLOUD-CLONE CORP.), Mouse TnT ELISA kit (#LS-F8830, LifeSpan BioSciences, Inc.), Mouse CK-MB ELISA kit (#MBS265235, MyBioSource), ATP ELISA kit (#MAK190-1KT, Sigma-Aldrich), Mouse SOD ELISA kit (#E4583, BioVision), Mouse glutathione ELISA kit (#MOEB2568, AssayGenie) and Mouse CAT ELISA kit (#EKU03008, Biomatik) were used according to the manufacturers' instructions to analyze the levels of the targeted proteins or enzymes.

### Co-immunoprecipitation

For co-immunoprecipitation, all steps were performed at 4 °C. Cells were harvested and then solubilized with immunoprecipitation buffer (150 mM NaCl, 10% glycerol, 20 mM Tris-HCl pH 7.4, 2 mM ethylenediaminetetraacetic acid, 0.5% Nonidet P-40, 0.5% Triton X-100 and complete protease inhibitor) for 1.5 hours. Insoluble components were removed via centrifugation at 12,000 × *g* for 10 minutes, and the supernatant was collected. One-tenth of the supernatant was removed and used as 'Input', while the rest was incubated with an anti-Flag M2 affinity gel (Sigma-Aldrich) at 4 °C overnight. The beads were washed six times with lysis buffer and then boiled in sodium dodecyl sulfate sample buffer to retrieve the proteins (immunoprecipitation products). Finally, the samples were analyzed using Western blotting.

### Molecular docking

For the docking analysis, we used the Schrödinger 2017-1 suite (Schrödinger Inc., New York, NY, USA). The crystal structures of DUSP1 and PHB2 were obtained from available sources and prepared for docking using the Protein Preparation Wizard workflow in the Maestro package. The binding site was chosen through the Grid Generation procedure. The prepared ligand was flexibly docked into the receptor using Glide (XP mode) with default settings. To determine the binding affinity between DUSP1 and PHB2, we employed the Microscale Thermophoresis methodology.

### Statistical analyses

All statistical analyses were conducted using GraphPad Prism 8 software. Data are presented as the mean ± standard deviation, and were compared using one-way or two-way analysis of variance. Statistical significance was set at p<0.05.

## Results

### DUSP1 overexpression sustains heart function after CRS-3

**Figure 1:**
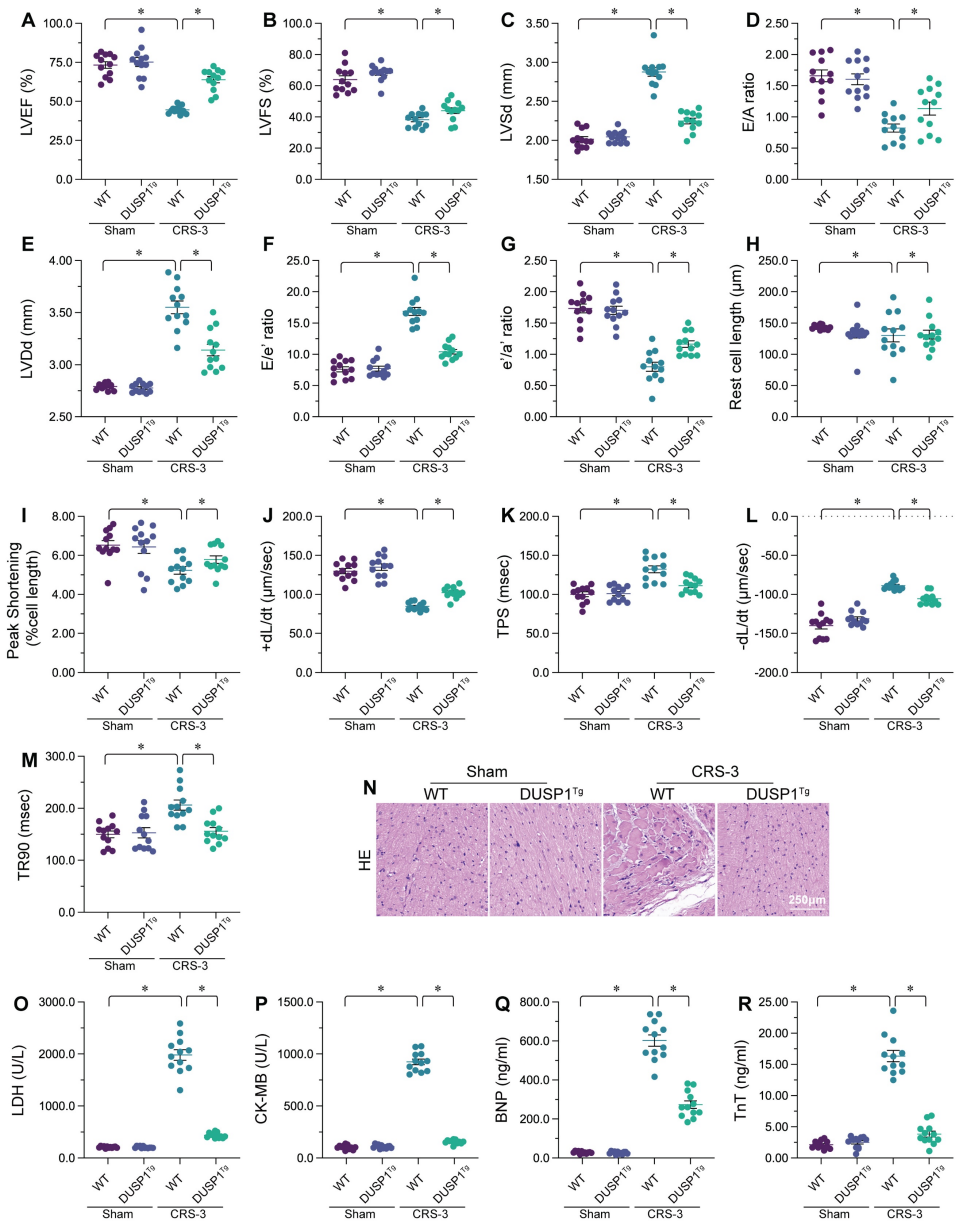
** DUSP1 overexpression sustains heart function after CRS-3.** DUSP1*^Tg^* and WT mice were subjected to 30 minutes of bilateral renal artery ischemia, followed by 72 hours of reperfusion to induce CRS-3. **A-G.** Echocardiography was used to evaluate heart function in DUSP1*^Tg^* and WT mice upon CRS-3. **H-M.** Single cardiomyocytes were isolated from DUSP1*^Tg^* and WT mice, and their contractile/relaxation capacities were recorded. **N.** H&E staining was used to observe the cardiac structure following CRS-3.** O-R.** Serum samples were collected from DUSP1*^Tg^* and WT mice, and the concentrations of TnT, CK-MB, BNP and LDH were determined using ELISAs. *p<0.05.

To explore the involvement of DUSP1 in CRS-3, we performed the CRS-3 operation on DUSP1 transgenic (DUSP1*^Tg^*) mice. We used echocardiography to compare the heart function of sham-operated mice, wild-type (WT) CRS-3 model mice and DUSP1*^Tg^* CRS-3 model mice (Figure 1A-G). Compared with the sham group, the WT CRS-3 group exhibited significantly reduced left ventricular ejection fraction (LVEF) and fractional shortening (FS) values, as well as impaired early to late ventricular filling velocity ratios (E/A) and early diastolic mitral inflow to early diastolic mitral annulus velocity ratios (E/e'). Interestingly, CRS-3 failed to induce heart dysfunction in DUSP1*^Tg^* mice (Figure 1A-G).

Subsequently, we isolated single cardiomyocytes from the mice to analyze their contractile/relaxation properties. As shown in Figure 1H-M, CRS-3 impaired cardiomyocyte contractile indexes such as the peak shortening (PS), maximal velocity of shortening (+dL/dt) and time to peak shortening (TPS). CRS-3 also diminished the cardiomyocyte relaxation capacity, as evidenced by the altered maximal velocity of relengthening (-dL/dt) and time to 90% relengthening (TR90). Interestingly, CRS-3 did not damage the mechanical properties of cardiomyocytes isolated from DUSP1*^Tg^* mice (Figure 1H-M).

We next examined structural changes in heart tissues from mice subjected to CRS-3. Histological analyses revealed myocardial fiber degradation and disorder following CRS-3 in WT mice, but not in DUSP1*^Tg^* mice (Figure 1N). Similarly, CRS-3 upregulated serum troponin T (TnT), lactate dehydrogenase (LDH), natriuretic peptide B (BNP) and creatine kinase (CK)-MB levels in WT mice, but not in DUSP1*^Tg^* mice (Figure 1O-R). In sum, CRS-3 downregulated DUSP1 in heart tissues, while DUSP1 overexpression suppressed CRS-3-induced myocardial dysfunction.

### DUSP1 overexpression reduces the cardiac inflammatory response, oxidative stress and mitochondrial injury during CRS-3

**Figure 2:**
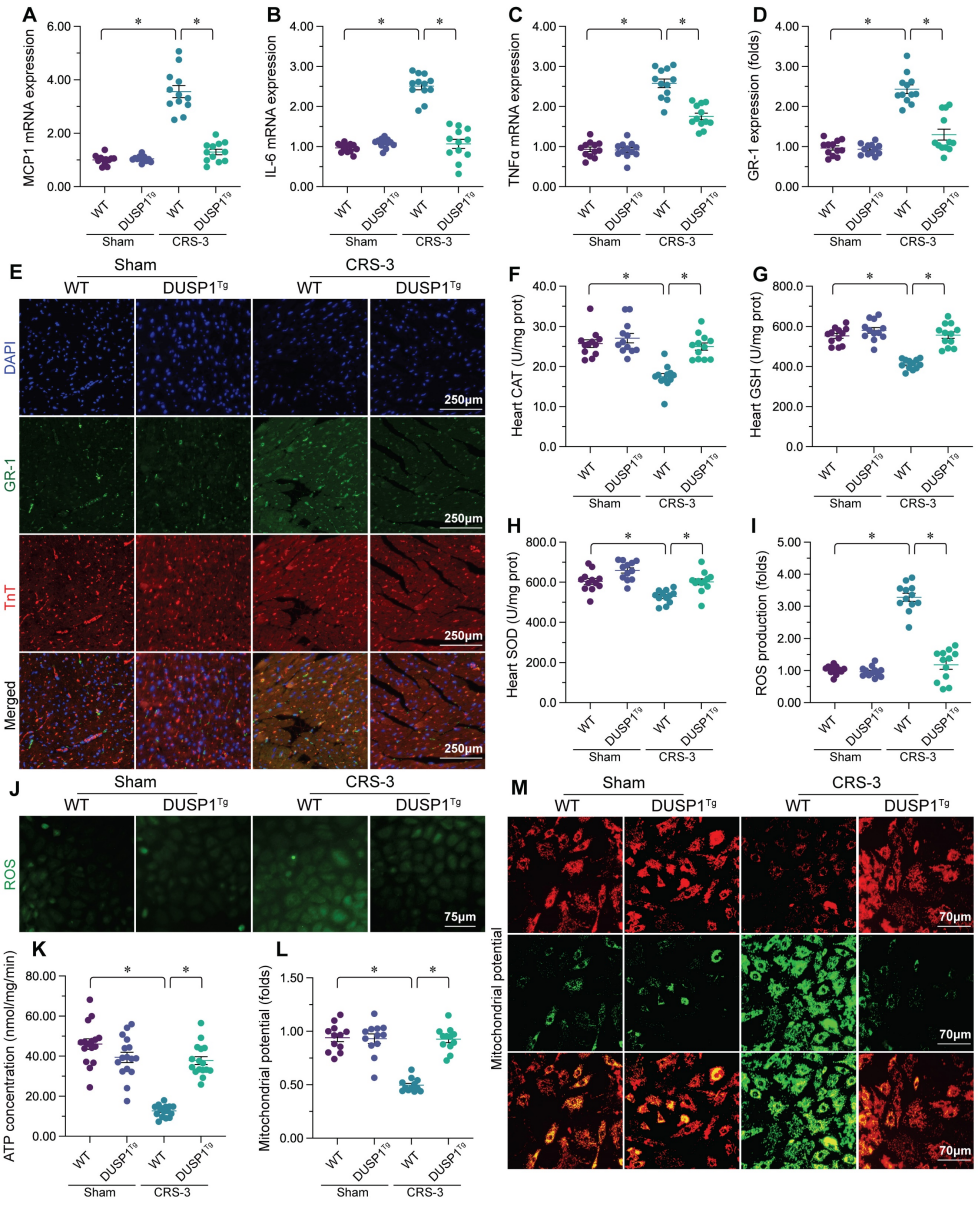
** DUSP1 reduces the inflammatory response, oxidative stress and mitochondrial injury in the heart during CRS-3.** DUSP1*^Tg^* and WT mice were subjected to 30 minutes of bilateral renal artery ischemia, followed by 72 hours of reperfusion to induce CRS-3. **A-C.** RNA was collected from DUSP1*^Tg^* and WT hearts, and qPCR was used to evaluate the transcription of *IL-6*, *TNFα* and* MCP1*. **D, E.** Double immunofluorescence staining was used to measure Gr-1-positive neutrophil accumulation in the myocardium. TnT was used to visualize myocardial fibers, and 4′,6-diamidino-2-phenylindole (DAPI) was used to stain the nuclei. **F-H.** ELISAs were used to evaluate the concentrations of glutathione (GSH), SOD and CAT in the heart. **I, J.** Cardiomyocytes were isolated from DUSP1*^Tg^* and WT mice, and ROS levels were determined using DCFH-DA. **K.** ATP production in the myocardium was determined with an ELISA. **L-M.** An immunofluorescence assay was used to determine the mitochondrial membrane potential in cardiomyocytes isolated from DUSP1*^Tg^* and WT mice after CRS-3. *p<0.05.

Our previous studies have indicated that CRS-3 is characterized by oxidative stress, the inflammatory response and mitochondrial injury in the heart 10. Thus, we asked whether DUSP1 overexpression could attenuate these pathological alterations in the presence of CRS-3. Quantitative real-time PCR (qPCR) analyses demonstrated that interleukin 6 (*IL-6*), tumor necrosis factor alpha (*TNFα*) and monocyte chemoattractant protein 1 (*MCP1*) levels were significantly elevated in heart tissues following CRS-3, whereas DUSP1 overexpression attenuated these changes (Figure 2A-C). Immunofluorescence staining showed that, as a result of increased inflammatory factor expression, Gr-1-positive neutrophil levels were elevated within the myocardium in CRS-3-treated WT mice, but not in DUSP1*^Tg^* mice (Figure 2D and E). These results confirmed that DUSP1 could inhibit the inflammatory response in the presence of CRS-3.

To evaluate oxidative stress in heart tissues from the different groups of mice, we performed enzyme-linked immunosorbent assays (ELISAs) to analyze the concentrations of glutathione (GSH), catalase (CAT) and superoxide dismutase (SOD). As shown in Figure 2F-H, glutathione, CAT and SOD activity levels were markedly downregulated in WT heart tissues upon CRS-3 treatment; however, DUSP1 overexpression restored glutathione, CAT and SOD levels in the myocardium. We then isolated cardiomyocytes from the mice, and used 2,7-dichlorodihydrofluorescein diacetate (DCFH-DA) staining to assess reactive oxygen species (ROS) production. Upon CRS-3 treatment, ROS levels were significantly elevated in WT cardiomyocytes, but were reduced to near-physiological levels in DUSP1*^Tg^* cardiomyocytes (Figure 2I and J). These data confirmed that DUSP1 overexpression could neutralize cardiac oxidative stress during CRS-3.

Mitochondrial injury often features reduced mitochondrial metabolism. An ELISA of total ATP production indicated that CRS-3 suppressed ATP synthesis in WT-derived cardiomyocytes, but not in DUSP1*^Tg^*-derived cardiomyocytes (Figure 2K). Since ATP metabolism highly depends on the mitochondrial membrane potential, we wondered whether DUSP1 overexpression influenced this parameter. The mitochondrial membrane potential was reduced upon CRS-3 treatment in WT cardiomyocytes, whereas this alteration was undetectable in DUSP1*^Tg^* cardiomyocytes (Figure 2L and M). Overall, DUSP1 preserved mitochondrial function in the heart during CRS-3.

### DUSP1 overexpression maintains mitochondrial quality control in the heart during CRS-3

**Figure 3:**
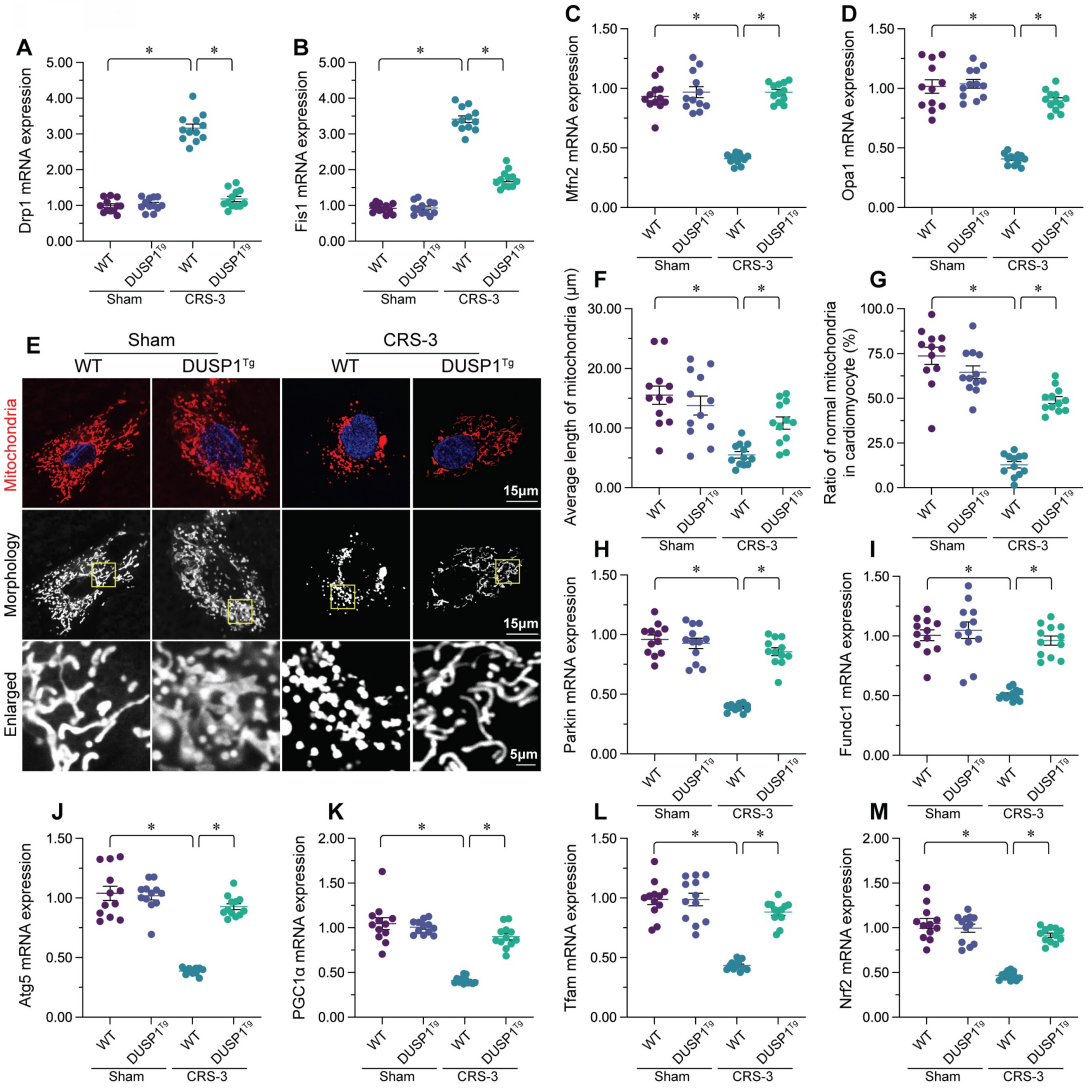
** DUSP1 overexpression maintains cardiac mitochondrial quality control during CRS-3.** DUSP1*^Tg^* and WT mice were subjected to 30 minutes of bilateral renal artery ischemia, followed by 72 hours of reperfusion to induce CRS-3. **A-D.** RNA was collected from DUSP1*^Tg^* and WT hearts, and qPCR was used to evaluate the transcription of *Drp1*, *Fis1*, *Mfn2* and *Opa1*. **E-G.** Immunofluorescence was used to stain the mitochondria of cardiomyocytes isolated from DUSP1*^Tg^* and WT mice. The average length of the mitochondria and the proportion of fragmented mitochondria were recorded. **H-J.** RNA was collected from DUSP1*^Tg^* and WT hearts, and qPCR was used to evaluate the transcription of *Parkin*, *Fundc1* and *Beclin1*. **K-M.** RNA was collected from DUSP1*^Tg^* and WT hearts, and qPCR was used to evaluate the transcription of *PGC1α*, *Nrf2* and *Tfam*. *p<0.05.

Mitochondrial quality control, including mitochondrial dynamics, mitophagy and mitochondrial biogenesis, is an endogenous protective mechanism that sustains mitochondrial function 15, 47, 48. Thus, we wondered whether DUSP1 protected cardiac mitochondria during CRS-3 by improving mitochondrial quality control. A qPCR analysis of mitochondrial dynamics proteins revealed that CRS-3 significantly upregulated dynamin-related protein 1 (*Drp1*) and mitochondrial fission 1 (*Fis1*) in heart tissues from WT mice, but not from DUSP1*^Tg^* mice (Figure 3A-D). On the other hand, mitochondrial fusion factors such as mitofusin 2 (*Mfn2*) and optic atrophy 1 (*Opa1*) were markedly downregulated following CRS-3 in WT heart tissues, but returned to near-normal levels in DUSP1*^Tg^* heart tissues (Figure 3A-D). Thus, CRS-3 activated mitochondrial fission and inhibited fusion in the heart, whereas DUSP1 overexpression normalized these processes. In accordance with these findings, immunofluorescence staining of cardiomyocyte mitochondria revealed that CRS-3 caused mitochondrial fragmentation in WT-derived cardiomyocytes (Figure 3E-G). However, the mitochondrial structure and network remained intact in DUSP1*^Tg^*-derived cardiomyocytes, despite treatment with CRS-3 (Figure 3E-G).

In addition to mitochondrial dynamics, we also noted that mitophagy was significantly suppressed by CRS-3 in WT heart tissues, as evidenced by reduced expression of *Parkin*, FUN14 domain-containing 1 (*Fundc1*) and *Beclin1* (Figure 3H-J). Interestingly, in DUSP1*^Tg^* heart tissues, CRS-3 failed to suppress mitophagy activity (Figure 3H-J).

The transcriptional process of mitochondrial biogenesis is highly regulated by peroxisome proliferator activated receptor gamma coactivator 1 alpha (PGC1α) and its downstream effectors - nuclear factor erythroid 2-related factor 2 (Nrf2) and transcription factor A, mitochondrial (Tfam). A qPCR analysis demonstrated that CRS-3 inhibited mitochondrial biogenesis gene expression in WT hearts, but not in DUSP1*^Tg^* hearts (Figure 3K-M). These data demonstrated that CRS-3 impaired cardiac mitochondrial quality control, while DUSP1 normalized it.

### DUSP1 binds to PHB2 to sustain its phosphorylation in the heart during CRS-3

**Figure 4:**
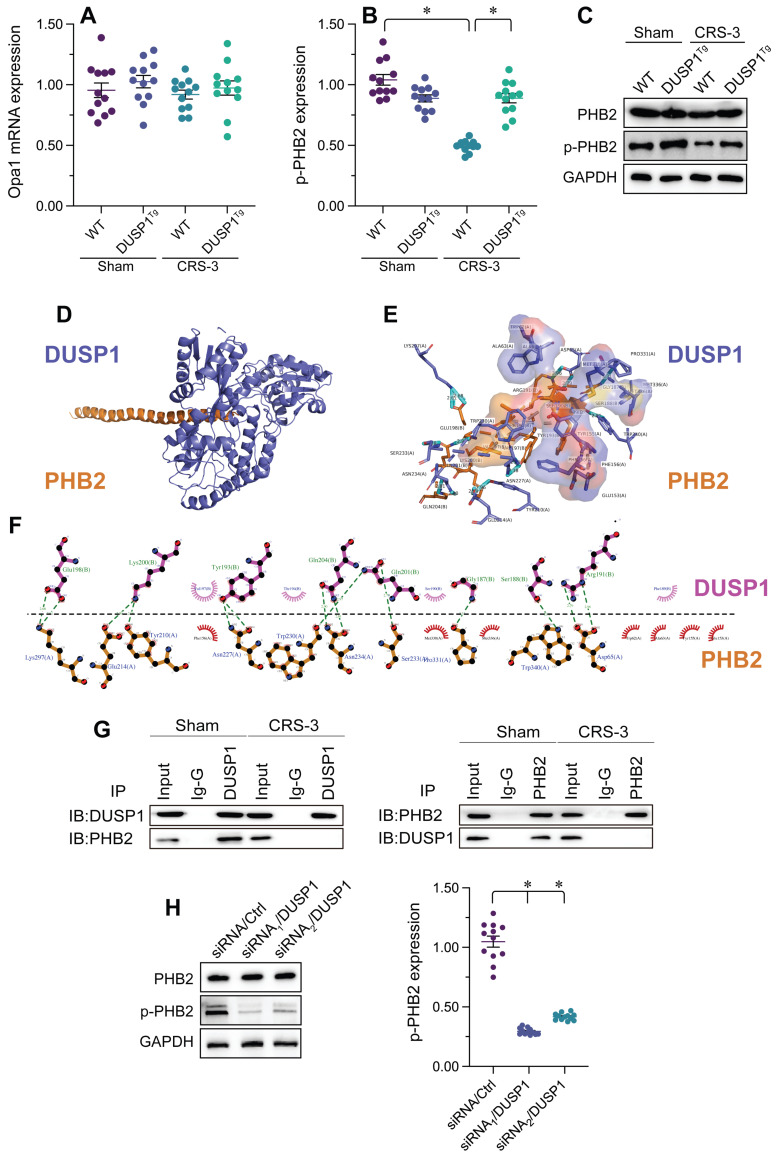
** DUSP1 binds to PHB2 to sustain its phosphorylation in the heart during CRS-3.** DUSP1*^Tg^* and WT mice were subjected to 30 minutes of bilateral renal artery ischemia, followed by 72 hours of reperfusion to induce CRS-3. **A.** RNA was collected from DUSP1*^Tg^* and WT hearts, and qPCR was used to evaluate the transcription of *PHB2*. **B, C.** Proteins were isolated from DUSP1*^Tg^* and WT hearts, and Western blotting was used to analyze PHB2 expression. **D, E.** Molecular docking analysis was used to evaluate the potential binding sites between DUSP1 and PHB2. **F, G.** Co-immunoprecipitation was used to analyze the crosslinks between DUSP1 and PHB2. **H, I.** HL-1 cells were transfected with siRNA against *DUSP1,* and the phosphorylation of PHB2 was determined via Western blotting. *p<0.05.

Recent studies have indicated that PHB2 promotes mitochondrial quality control by suppressing mitochondrial fission and inducing mitophagy. Interestingly, in heart tissues from CRS-3 model mice, the transcription of *PHB2* was unaltered compared with the control group (Figure 4A), so total PHB2 levels remained constant; however, PHB2 phosphorylation was attenuated following CRS-3 (Figure 4B and C). PHB2 phosphorylation in the heart returned to near-normal levels in response to DUSP1 overexpression, suggesting that DUSP1 governs the phosphorylation status of PHB2 in the setting of CRS-3 (Figure 4B and C).

To determine how DUSP1 sustains PHB2 phosphorylation, we analyzed the possibility of a protein-protein interaction between the two. We first performed a molecular docking assay, which revealed potential binding sites between DUSP1 and PHB2 (Figure 4D-F). Co-immunoprecipitation experiments further confirmed the presence of crosslinks between DUSP1 and PHB2 (Figure 4G). To verify that DUSP1 is required to preserve PHB2 phosphorylation, we transfected HL-1 cells with siRNA against *DUSP1* under physiological conditions. Knocking down *DUSP1* reduced PHB2 phosphorylation (Figure 4H). These results demonstrated that DUSP1 binds directly to PHB2 to maintain PHB2 phosphorylation, while the downregulation of DUSP1 during CRS-3 contributes to PHB2 dephosphorylation.

### Transgenic mice expressing a *Phb2^S91D^* phosphorylation mutant gene are resistant to CRS-3

**Figure 5:**
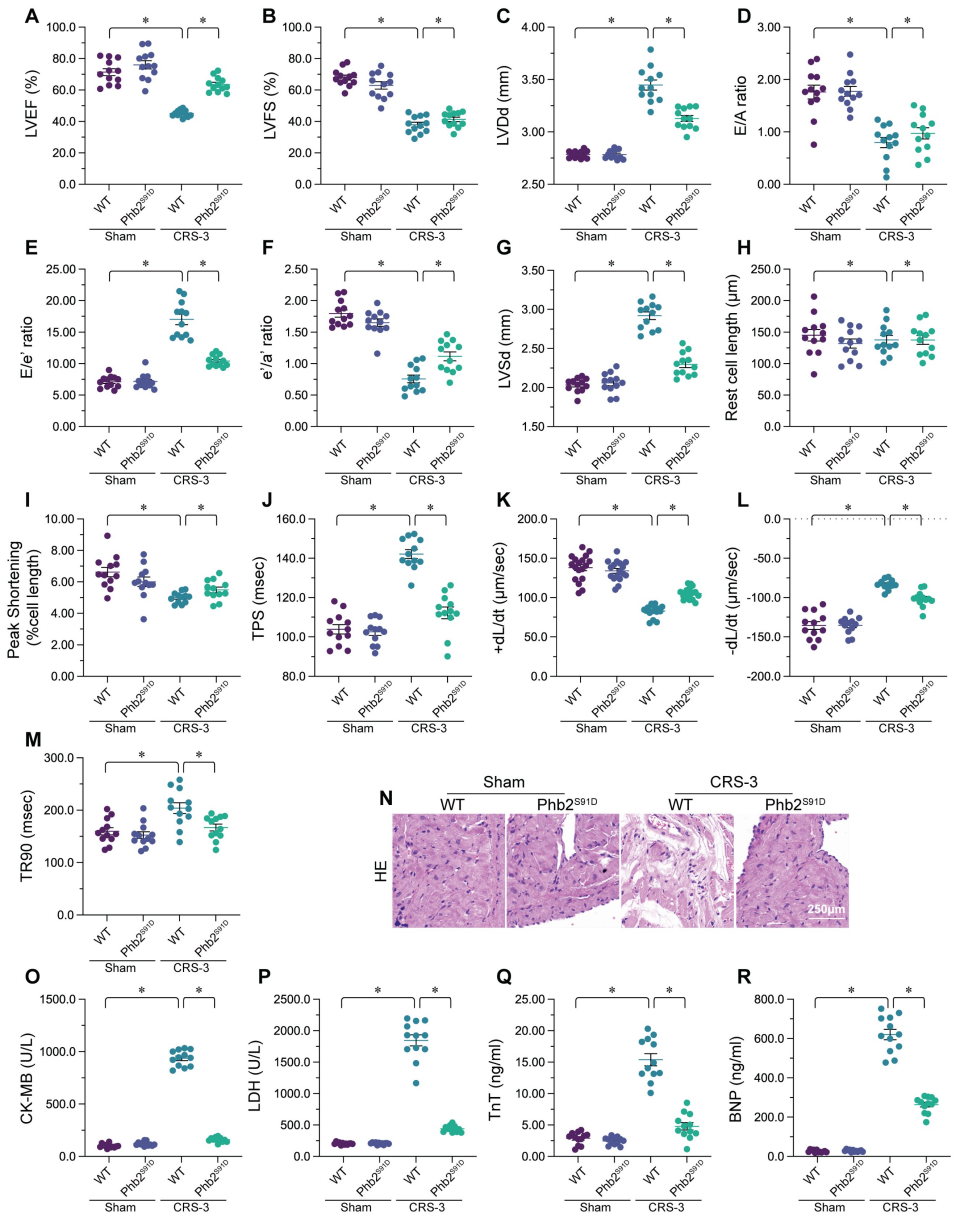
** Transgenic mice expressing a *Phb2^S91D^* phosphorylation mutant gene are resistant to CRS-3.** *Phb2^S91D^* knock-in and WT mice were subjected to 30 minutes of bilateral renal artery ischemia, followed by 72 hours of reperfusion to induce CRS-3. **A-G.** Echocardiography was used to evaluate heart function in *Phb2^S91D^* knock-in and WT mice upon CRS-3. **H-M.** Single cardiomyocytes were isolated from *Phb2^S91D^* knock-in and WT mice, and their contractile/relaxation capacities were recorded. **N.** H&E staining was used to observe the cardiac structure following CRS-3.** O-R.** Serum samples were collected from *Phb2^S91D^* knock-in and WT mice, and the concentrations of TnT, CK-MB, BNP and LDH were determined with ELISAs. *p<0.05.

To test whether PHB2 dephosphorylation is a pathological factor that impairs cardiac mitochondrial quality control and promotes myocardial dysfunction in CRS-3, we used transgenic knock-in mice carrying the *Phb2^S91D^* variant on a C57BL/6 background, as we previously described 36. The LVEF, FS, E/A, LVDd, LVSd, and E/e' values were also improved in *Phb2^S91D^* mice compared with WT mice following CRS-3 (Figure 5A-G).

Subsequently, we isolated single cardiomyocytes from the mice to analyze their contractile/relaxation properties. As shown in Figure 5H-M, the contractile indexes (PS, +dL/dt and TPS) that were disrupted by CRS-3 in WT cardiomyocytes were restored to near-normal levels in *Phb2^S91D^* cardiomyocytes. Likewise, CRS-3 impaired the relaxation capacity of WT cardiomyocytes, but not of *Phb2^S91D^* cardiomyocytes (Figure 5H-M).

We then performed a histological analysis, which showed that CRS-3 failed to induce myocardial fiber degradation or disorder in *Phb2^S91D^* heart tissues (Figure 5N). Similarly, although CRS-3 upregulated TnT, LDH, BNP and CK-MB in WT serum samples, these changes were reversed in *Phb2^S91D^* mice (Figure 5O-R). Thus, preserving PHB2 phosphorylation seemed to protect the heart against CRS-3-induced myocardial injury.

### Enhancing PHB2 phosphorylation attenuates mitochondrial quality control disorder, oxidative stress and the inflammatory response in the heart following CRS-3

**Figure 6:**
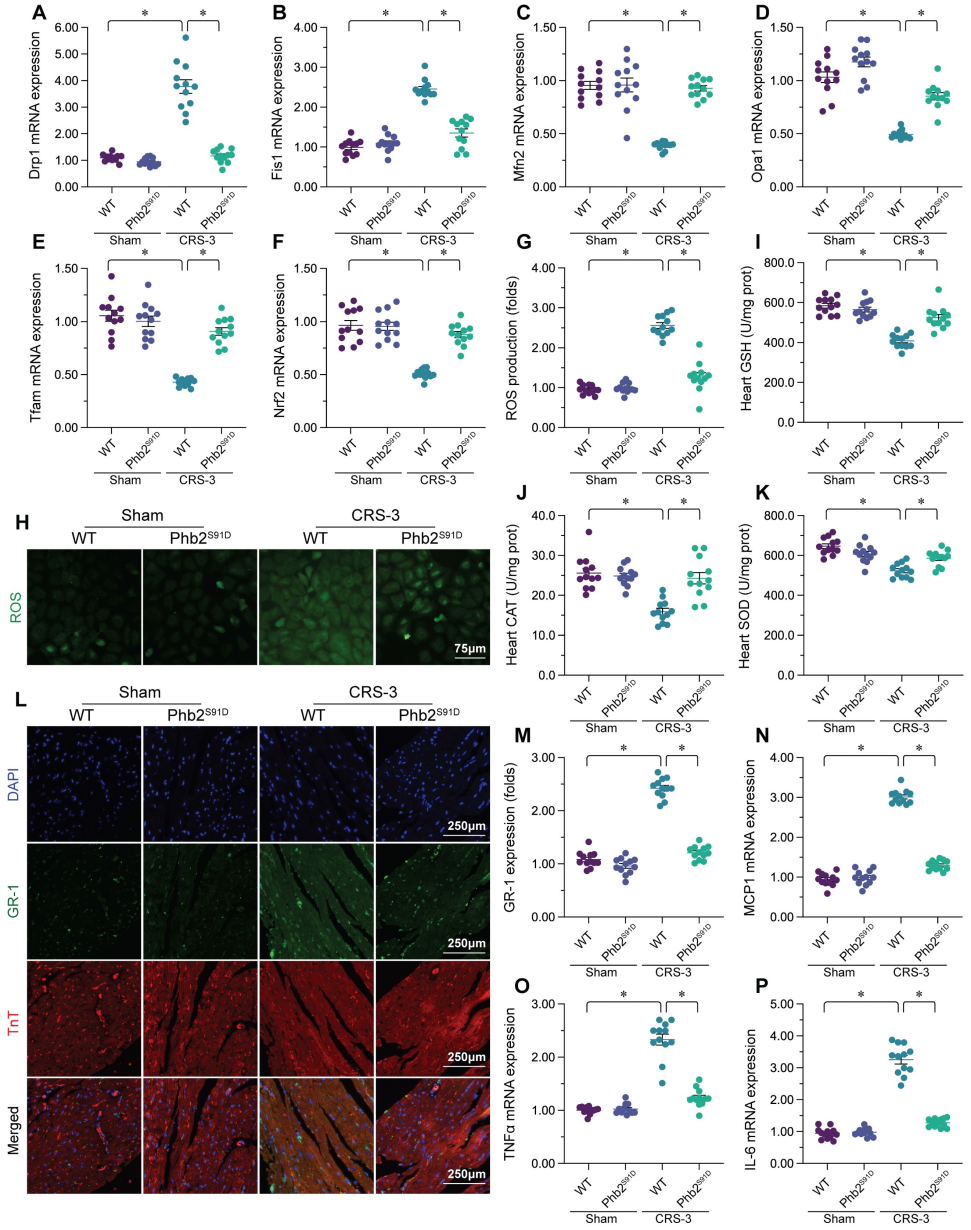
** Enhancing PHB2 phosphorylation attenuates cardiac mitochondrial quality control disorder, oxidative stress and the inflammatory response during CRS-3.** *Phb2^S91D^* knock-in and WT mice were subjected to 30 minutes of bilateral renal artery ischemia, followed by 72 hours of reperfusion to induce CRS-3. **A-F.** RNA was collected from *Phb2^S91D^* knock-in and WT hearts, and qPCR was used to evaluate the transcription of *Drp1*, *Fis1*, *Mfn2*, *Opa1*, *Nrf2* and *Tfam*. **G, H.** Cardiomyocytes were isolated from *Phb2^S91D^* knock-in and WT mice, and ROS levels were determined using DCFH-DA.** I-K.** ELISAs were used to evaluate the concentrations of glutathione (GSH), SOD and CAT in the heart. **L, M.** Double immunofluorescence staining was used to measure Gr-1-positive neutrophil accumulation in the myocardium. TnT was used to visualize myocardial fibers, and DAPI was used to stain the nuclei. **N-P.** RNA was collected from *Phb2^S91D^* knock-in and WT hearts, and qPCR was used to evaluate the transcription of *IL-6*, *TNFα* and *MCP1*. *p<0.05.

We then assessed whether PHB2 phosphorylation protected the heart during CRS-3 by altering mitochondrial quality control, oxidative stress or the inflammatory response. We found that CRS-3 upregulated *Drp1*/*Fis1* and downregulated *Mfn2*/*Opa1* in WT heart tissues, while these changes were significantly suppressed in *Phb2^S91D^* heart tissues (Figure 6A-D). Similarly, CRS-3 significantly inhibited cardiac *Nrf2* and *Tfam* expression in WT mice, but not in *Phb2^S91D^* mice (Figure 6E-F).

*In vitro*, immunofluorescence staining indicated that ROS generation was augmented in WT cardiomyocytes upon CRS-3 exposure, while it was reduced in *Phb2^S91D^* cardiomyocytes in the setting of CRS-3 (Figure 6G and H). ELISAs demonstrated that CRS-3 reduced the activity of glutathione, CAT and SOD in WT heart, but not in *Phb2^S91D^* heart (Figure 6I-K).

Double immunofluorescence staining of the myocardium revealed that CRS-3 induced Gr-1-positive neutrophil accumulation within WT heart tissues, while this inflammatory response was undetectable in *Phb2^S91D^* mice (Figure 6L and M). Accordingly, CRS-3 failed to upregulate pro-inflammatory genes in heart tissues from *Phb2^S91D^* mice (Figure 6N-P). These data illustrated that PHB2 phosphorylation prevented CRS-3-related myocardial injury by improving mitochondrial quality control, reducing oxidative stress and repressing the inflammatory response in the heart.

## Discussion

Under normal conditions, an organ-organ feedback system maintains the balance between the heart and the kidneys. The heart perfuses the organs by continuously pumping fresh blood, while the kidneys reabsorb water from urine and regulate the blood volume through neuroendocrine control, thus decisively influencing the cardiac output. However, under pathological conditions, dysfunction in one organ can lead to dysfunction in the other, either acutely or chronically. CRS refers to abnormalities in the heart-kidney interaction 49, and CRS-3 refers to acute cardiac dysfunction caused by acute kidney injury.

Compared with other types of CRS, CRS-3 has been a neglected area of research, so many academic viewpoints and experimental hypotheses regarding its pathogenesis remain unverified. Among these research conclusions, the widely accepted pathogenic mechanism of CRS-3 is that acute kidney injury causes hemodynamic abnormalities that lead to cardiac dysfunction. Specifically, when the kidneys are injured, inadequate renal perfusion activates the renin-angiotensin-aldosterone system, stimulates the sympathetic nervous system and promotes vasopressin secretion, leading to fluid retention, an increased preload and exacerbated pump dysfunction 2, 50. However, cardiac preload and afterload abnormalities and sympathetic nervous system activation can only partially explain the pathophysiological changes in type-3 CRS 3. In clinical practice, the use of vasoactive drugs has not completely improved the symptoms of CRS-3 patients 51, 52, suggesting that non-hemodynamic factors may also be involved in the pathogenesis of this disease 53, 54. Among these factors, inflammatory response activation, myocardial substrate metabolism conversion, mitochondrial fission and imbalanced ROS/nitric oxide production have been proposed as potential pathophysiological contributors to CRS-3 49, 55-57. However, there is still insufficient research to explain the relationships among these molecular events or determine their common upstream signaling pathways systematically.

In the present study, we found that CRS-3-induced myocardial dysfunction was associated with defective mitochondrial quality control, including increased mitochondrial fission, reduced mitochondrial fusion, diminished mitophagy and suppressed mitochondrial biogenesis in the heart. Abnormal mitochondrial quality control has been reported as a pathological factor in several cardiovascular diseases, including cardiac ischemia/reperfusion injury 18, 58, septic cardiomyopathy 17, 59 and diabetic cardiomyopathy 14, 60. Increased mitochondrial fission and reduced mitochondrial fusion are thought to activate mitochondrial apoptosis by two main mechanisms 61, 62. First, an imbalance between mitochondrial fission and fusion promotes the formation of mitochondrial fragments containing broken or incomplete mitochondrial DNA, so the mitochondria-encoded respiratory complex enzymes are transcriptionally downregulated 61. This accelerates mitochondrial ROS production, which induces cardiolipin oxidation and cytochrome c release from mitochondria into the cytoplasm 61, 63. Second, excessive fission and defective fusion induce the disassociation of hexokinase-II from mitochondria, thus promoting mitochondrial permeability transition pore opening 64. Through these two pathways, mitochondrial dynamics disorder not only induces mitochondrial dysfunction, but also promotes cardiomyocyte apoptosis.

In addition to altered mitochondrial dynamics, impaired mitophagy and mitochondrial biogenesis have been associated with reduced mitochondrial turnover 38, 65 and the accumulation of poorly-structured mitochondria within cardiomyocytes. Although previous studies have described the involvement of mitochondria in myocardial dysfunction during CRS-3 8, 10, 11, this is the first study to delineate the molecular pathway by which both mitochondrial function and structure are disrupted during CRS-3. Our findings suggest that normalizing mitochondrial quality control is critical for preserving cardiomyocyte mitochondrial function and cell viability in the setting of CRS-3.

Importantly, our study revealed that DUSP1 downregulation and PHB2 dephosphorylation are the molecular mechanisms accounting for mitochondrial quality control disorder in the heart upon CRS-3. Ample studies have elucidated the cardioprotective effects of DUSP1. DUSP1 deficiency was reported to exacerbate cardiac ischemia/reperfusion injury by activating mitochondrial fission through mitochondrial fission factor in a manner dependent on the c-Jun N-terminal kinase pathway 26. DUSP1 overexpression promoted valosin-containing protein dephosphorylation in the heart, thus improving mitochondrial quality control, mitochondrial integrity and heart function during septic cardiomyopathy 16, 27. In the present study, we found that DUSP1 bound directly to PHB2 to maintain its phosphorylation, while CRS-3 stress downregulated DUSP1, thereby reducing PHB2 phosphorylation as an initial signal of abnormal mitochondrial quality control in the heart. Interestingly, previous studies have indicated that dephosphorylated PHB2 is associated with mitochondrial dysfunction or cell apoptosis 35, 36. We found that sustaining PHB2 phosphorylation *in vivo* enhanced the resistance of the myocardium to CRS-3 by reducing the inflammatory response, inhibiting oxidative stress and improving mitochondrial quality control, ultimately improving heart function.

This study had several limitations. First, additional *in vitro* data are necessary to illuminate the protein-protein interaction between DUSP1 and PHB2. Second, it remains unknown how acute kidney injury downregulates DUSP1 in the myocardium. It will be interesting to determine whether neuromodulators, serum factors/cytokines or microRNAs allow the injured kidneys to communicate with the myocardium in the pathogenesis of CRS-3.

In conclusion, our results demonstrated that CRS-3 induced heart failure by suppressing cardiac DUSP1 expression. DUSP1 overexpression effectively maintained heart function by suppressing the inflammatory response, inhibiting oxidative stress and improving mitochondrial quality control. DUSP1 bound directly to PHB2 to prevent its dephosphorylation; thus, overexpressing DUSP1 or enhancing PHB2 phosphorylation reduced the vulnerability of the heart to CRS-3. Based on our findings, therapeutic approaches to enhance DUSP1 expression, PHB2 phosphorylation or mitochondrial quality control could improve the treatment of CRS-3-related myocardial depression.

## Supplementary Material

Supplementary tables.

## References

[1] Zannad F, Rossignol P (2018). Cardiorenal Syndrome Revisited. Circulation.

[2] Rangaswami J, Bhalla V, Blair JEA, Chang TI, Costa S, Lentine KL (2019). Cardiorenal Syndrome: Classification, Pathophysiology, Diagnosis, and Treatment Strategies: A Scientific Statement From the American Heart Association. Circulation.

[3] Costanzo MR (2020). The Cardiorenal Syndrome in Heart Failure. Heart Fail Clin.

[4] Patschan D, Marahrens B, Jansch M, Patschan S, Ritter O (2022). Experimental Cardiorenal Syndrome Type 3: What Is Known so Far?. J Clin Med Res.

[5] Okpara R, Pena C, Nugent K (2022). Cardiorenal Syndrome Type 3 Review. Cardiol Rev.

[6] Bagshaw SM, Hoste EA, Braam B, Briguori C, Kellum JA, McCullough PA (2013). Cardiorenal syndrome type 3: pathophysiologic and epidemiologic considerations. Contrib Nephrol.

[7] Clementi A, Virzì GM, Brocca A, de Cal M, Pastori S, Clementi M (2015). Advances in the pathogenesis of cardiorenal syndrome type 3. Oxid Med Cell Longev.

[8] Wang J, Sun X, Wang X, Cui S, Liu R, Liu J (2021). Grb2 Induces Cardiorenal Syndrome Type 3: Roles of IL-6, Cardiomyocyte Bioenergetics, and Akt/mTOR Pathway. Front Cell Dev Biol.

[9] Di Lullo L, Bellasi A, Barbera V, Cozzolino M, Russo D, De Pascalis A (2016). [Acute Kidney Injury, Type - 3 cardiorenal syndrome, Biomarkers, Renal Replacement Therapy]. G Ital Nefrol.

[10] Wang J, Toan S, Li R, Zhou H (2020). Melatonin fine-tunes intracellular calcium signals and eliminates myocardial damage through the IP3R/MCU pathways in cardiorenal syndrome type 3. Biochem Pharmacol.

[11] Cai C, Wu F, Zhuang B, Ou Q, Peng X, Shi N (2022). Empagliflozin activates Wnt/β-catenin to stimulate FUNDC1-dependent mitochondrial quality surveillance against type-3 cardiorenal syndrome. Mol Metab.

[12] Ramachandra CJA, Hernandez-Resendiz S, Crespo-Avilan GE, Lin YH, Hausenloy DJ (2020). Mitochondria in acute myocardial infarction and cardioprotection. EBioMedicine.

[13] Zhu H, Toan S, Mui D, Zhou H (2021). Mitochondrial quality surveillance as a therapeutic target in myocardial infarction. Acta Physiol (Oxf).

[14] Chang X, Li Y, Cai C, Wu F, He J, Zhang Y (2022). Mitochondrial quality control mechanisms as molecular targets in diabetic heart. Metabolism.

[15] Chang X, Toan S, Li R, Zhou H (2022). Therapeutic strategies in ischemic cardiomyopathy: Focus on mitochondrial quality surveillance. EBioMedicine.

[16] Zhu H, Wang J, Xin T, Chen S, Hu R, Li Y (2023). DUSP1 interacts with and dephosphorylates VCP to improve mitochondrial quality control against endotoxemia-induced myocardial dysfunction. Cell Mol Life Sci.

[17] Zhou H, Dai Z, Li J, Wang J, Zhu H, Chang X (2023). TMBIM6 prevents VDAC1 multimerization and improves mitochondrial quality control to reduce sepsis-related myocardial injury. Metabolism.

[18] Zhu H, Tan Y, Du W, Li Y, Toan S, Mui D (2021). Phosphoglycerate mutase 5 exacerbates cardiac ischemia-reperfusion injury through disrupting mitochondrial quality control. Redox Biol.

[19] Yu LM, Dong X, Xue XD, Xu S, Zhang X, Xu YL (2021). Melatonin attenuates diabetic cardiomyopathy and reduces myocardial vulnerability to ischemia-reperfusion injury by improving mitochondrial quality control: Role of SIRT6. J Pineal Res.

[20] Chen L, Qin Y, Liu B, Gao M, Li A, Li X (2022). PGC-1α-Mediated Mitochondrial Quality Control: Molecular Mechanisms and Implications for Heart Failure. Front Cell Dev Biol.

[21] Li G, Yang J, Yang C, Zhu M, Jin Y, McNutt MA (2018). PTENα regulates mitophagy and maintains mitochondrial quality control. Autophagy.

[22] Lang R, Hammer M, Mages J (2006). DUSP meet immunology: dual specificity MAPK phosphatases in control of the inflammatory response. J Immunol.

[23] Celaya AM, Sánchez-Pérez I, Bermúdez-Muñoz JM, Rodríguez-de la Rosa L, Pintado-Berninches L, Perona R (2019). Deficit of mitogen-activated protein kinase phosphatase 1 (DUSP1) accelerates progressive hearing loss. Elife.

[24] Korhonen R, Turpeinen T, Taimi V, Nieminen R, Goulas A, Moilanen E (2011). Attenuation of the acute inflammatory response by dual specificity phosphatase 1 by inhibition of p38 MAP kinase. Mol Immunol.

[25] Wang J, Zhou JY, Kho D, Reiners JJ Jr, Wu GS (2016). Role for DUSP1 (dual-specificity protein phosphatase 1) in the regulation of autophagy. Autophagy.

[26] Jin Q, Li R, Hu N, Xin T, Zhu P, Hu S (2018). DUSP1 alleviates cardiac ischemia/reperfusion injury by suppressing the Mff-required mitochondrial fission and Bnip3-related mitophagy via the JNK pathways. Redox Biol.

[27] Tan Y, Zhang Y, He J, Wu F, Wu D, Shi N (2022). Dual specificity phosphatase 1 attenuates inflammation-induced cardiomyopathy by improving mitophagy and mitochondrial metabolism. Mol Metab.

[28] Qi A, Lamont L, Liu E, Murray SD, Meng X, Yang S (2023). Essential Protein PHB2 and Its Regulatory Mechanisms in Cancer. Cells.

[29] Yan C, Gong L, Chen L, Xu M, Abou-Hamdan H, Tang M (2020). PHB2 (prohibitin 2) promotes PINK1-PRKN/Parkin-dependent mitophagy by the PARL-PGAM5-PINK1 axis. Autophagy.

[30] Wang K, Long B, Zhou LY, Liu F, Zhou QY, Liu CY (2014). CARL lncRNA inhibits anoxia-induced mitochondrial fission and apoptosis in cardiomyocytes by impairing miR-539-dependent PHB2 downregulation. Nat Commun.

[31] Wang J, Zhu P, Li R, Ren J, Zhang Y, Zhou H (2020). Bax inhibitor 1 preserves mitochondrial homeostasis in acute kidney injury through promoting mitochondrial retention of PHB2. Theranostics.

[32] Li S, Liu M, Chen J, Chen Y, Yin M, Zhou Y (2023). L-carnitine alleviates cardiac microvascular dysfunction in diabetic cardiomyopathy by enhancing PINK1-Parkin-dependent mitophagy through the CPT1a-PHB2-PARL pathways. Acta Physiol (Oxf).

[33] Yang M, Abudureyimu M, Wang X, Zhou Y, Zhang Y, Ren J (2023). PHB2 ameliorates Doxorubicin-induced cardiomyopathy through interaction with NDUFV2 and restoration of mitochondrial complex I function. Redox Biol.

[34] Bertolin G, Alves-Guerra MC, Cheron A, Burel A, Prigent C, Le Borgne R (2021). Mitochondrial Aurora kinase A induces mitophagy by interacting with MAP1LC3 and Prohibitin 2. Life Sci Alliance.

[35] Ross JA, Robles-Escajeda E, Oaxaca DM, Padilla DL, Kirken RA (2017). The prohibitin protein complex promotes mitochondrial stabilization and cell survival in hematologic malignancies. Oncotarget.

[36] Zou RJ, Tao J, He J, Wang CJ, Tan ST, Xia Y (2022). PGAM5-Mediated PHB2 Dephosphorylation Contributes to Diabetic Cardiomyopathy by Disrupting Mitochondrial Quality Surveillance. RESEARCH. 2022.

[37] Zhou H, Zhu P, Wang J, Toan S, Ren J (2019). DNA-PKcs promotes alcohol-related liver disease by activating Drp1-related mitochondrial fission and repressing FUNDC1-required mitophagy. Signal transduction and targeted therapy.

[38] Zhou H, Zhu P, Wang J, Zhu H, Ren J, Chen Y (2018). Pathogenesis of cardiac ischemia reperfusion injury is associated with CK2alpha-disturbed mitochondrial homeostasis via suppression of FUNDC1-related mitophagy. Cell Death Differ.

[39] Zhou H, Toan S, Zhu P, Wang J, Ren J, Zhang Y (2020). DNA-PKcs promotes cardiac ischemia reperfusion injury through mitigating BI-1-governed mitochondrial homeostasis. Basic Res Cardiol.

[40] Yao Y, Zhu P, Xu N, Jiang L, Tang XF, Song Y (2022). Effects of chronic obstructive pulmonary disease on long-term prognosis of patients with coronary heart disease post-percutaneous coronary intervention. J Geriatr Cardiol.

[41] Montero S, Abrams D, Ammirati E, Huang F, Donker DW, Hekimian G (2022). Fulminant myocarditis in adults: a narrative review. J Geriatr Cardiol.

[42] Huang BT, Yang L, Yang BS, Huang FY, Xiao QF, Pu XB (2022). Relationship of body fat and left ventricular hypertrophy with the risk of all-cause death in patients with coronary artery disease. J Geriatr Cardiol.

[43] Wang J, Zhu P, Li R, Ren J, Zhou H (2020). Fundc1-dependent mitophagy is obligatory to ischemic preconditioning-conferred renoprotection in ischemic AKI via suppression of Drp1-mediated mitochondrial fission. Redox Biol.

[44] Luo FY, Bai YP, Bu HS (2022). Protein quality control systems in hypertrophic cardiomyopathy: pathogenesis and treatment potential. J Geriatr Cardiol.

[45] Mene-Afejuku TO, Jeyashanmugaraja GP, Hoq M, Ola O, Shah AJ (2022). Determinants of mortality among seniors acutely readmitted for heart failure: racial disparities and clinical correlations. J Geriatr Cardiol.

[46] Hang PZ, Li PF, Liu J, Li FF, Chen TT, Pan Y (2022). Small-molecule 7,8-dihydroxyflavone counteracts compensated and decompensated cardiac hypertrophy via AMPK activation. J Geriatr Cardiol.

[47] Sun D, Wang J, Toan S, Muid D, Li R, Chang X (2022). Molecular mechanisms of coronary microvascular endothelial dysfunction in diabetes mellitus: focus on mitochondrial quality surveillance. Angiogenesis.

[48] Zhou H, Ren J, Toan S, Mui D (2021). Role of mitochondrial quality surveillance in myocardial infarction: From bench to bedside. Ageing Res Rev.

[49] Kumar U, Wettersten N, Garimella PS (2019). Cardiorenal Syndrome: Pathophysiology. Cardiol Clin.

[50] Bonanad C, Fernández-Olmo R, García-Blas S, Alarcon JA, Díez-Villanueva P, Mansilla CR (2022). Cardiovascular prevention in elderly patients. J Geriatr Cardiol.

[51] Vandenberghe W, Hoste EA (2014). Acute kidney injury survivors should have long-term follow-up. Crit Care.

[52] Cai JJ, Liu Y, Wang J, Wang JX, Wang Y, Xu SB (2022). Lactobacillus levels and prognosis of patients with acute myocardial infarction. J Geriatr Cardiol.

[53] Ronco C, Bellasi A, Di Lullo L (2018). Cardiorenal Syndrome: An Overview. Adv Chronic Kidney Dis.

[54] González-Colaço Harmand M, García-Sanz MDM, Agustí A, Prada-Arrondo PC, Domínguez-Rodríguez A, Grandal-Leirós B (2022). Review on the management of cardiovascular risk factors in the elderly. J Geriatr Cardiol.

[55] Savira F, Magaye R, Liew D, Reid C, Kelly DJ, Kompa AR (2020). Cardiorenal syndrome: Multi-organ dysfunction involving the heart, kidney and vasculature. Br J Pharmacol.

[56] Wang J, Zhang W, Wu L, Mei Y, Cui S, Feng Z (2020). New insights into the pathophysiological mechanisms underlying cardiorenal syndrome. Aging (Albany NY).

[57] Barrionuevo-Sánchez MI, Ariza-Solé A, Ortiz-Berbel D, González-Costello J, Gómez-Hospital JA, Lorente V (2022). Usefulness of Impella support in different clinical settings in cardiogenic shock. J Geriatr Cardiol.

[58] Zou R, Shi W, Qiu J, Zhou N, Du N, Zhou H (2022). Empagliflozin attenuates cardiac microvascular ischemia/reperfusion injury through improving mitochondrial homeostasis. Cardiovasc Diabetol.

[59] Zou R, Tao J, Qiu J, Lu H, Wu J, Zhu H (2022). DNA-PKcs promotes sepsis-induced multiple organ failure by triggering mitochondrial dysfunction. J Adv Res.

[60] Cai C, Wu F, He J, Zhang Y, Shi N, Peng X (2022). Mitochondrial quality control in diabetic cardiomyopathy: from molecular mechanisms to therapeutic strategies. International Journal of Biological Sciences.

[61] Zhou H, Hu S, Jin Q, Shi C, Zhang Y, Zhu P (2017). Mff-Dependent Mitochondrial Fission Contributes to the Pathogenesis of Cardiac Microvasculature Ischemia/Reperfusion Injury via Induction of mROS-Mediated Cardiolipin Oxidation and HK2/VDAC1 Disassociation-Involved mPTP Opening. J Am Heart Assoc.

[62] Zhou H, Zhang Y, Hu S, Shi C, Zhu P, Ma Q (2017). Melatonin protects cardiac microvasculature against ischemia/reperfusion injury via suppression of mitochondrial fission-VDAC1-HK2-mPTP-mitophagy axis. J Pineal Res.

[63] Zhou H, Shi C, Hu S, Zhu H, Ren J, Chen Y (2018). BI1 is associated with microvascular protection in cardiac ischemia reperfusion injury via repressing Syk-Nox2-Drp1-mitochondrial fission pathways. Angiogenesis.

[64] Wu B, Luo H, Zhou X, Cheng CY, Lin L, Liu BL (2017). Succinate-induced neuronal mitochondrial fission and hexokinase II malfunction in ischemic stroke: Therapeutical effects of kaempferol. Biochim Biophys Acta Mol Basis Dis.

[65] Ma T, Huang X, Zheng H, Huang G, Li W, Liu X (2021). SFRP2 Improves Mitochondrial Dynamics and Mitochondrial Biogenesis, Oxidative Stress, and Apoptosis in Diabetic Cardiomyopathy. Oxid Med Cell Longev.

