# Supplementary material for: Dual-specificity phosphatase 1 interacts with prohibitin 2 to improve mitochondrial quality control against type-3 cardiorenal syndrome
Source: Int J Med Sci. 2024 Jan 19;21(3):547–61. doi: 10.7150/ijms.90484 (PMC10845262; doi:10.7150/ijms.90484)
Supplement: Supplementary file 1 — Supplementary tables. [file ijmsv21p0547s1.pdf]

Supplementary Material

Table S5:

DUSP1 antibody	Abcam	#228987
PHB2	Abcam	#75766
GAPDH	Abcam	#9485
